# Polyvalent therapeutic vaccine for type 2 diabetes mellitus: Immunoinformatics approach to study co-stimulation of cytokines and GLUT1 receptors

**DOI:** 10.1186/s12860-020-00279-w

**Published:** 2020-07-23

**Authors:** Syed Aun Muhammad, Hiba Ashfaq, Sidra Zafar, Fahad Munir, Muhammad Babar Jamshed, Jake Chen, Qiyu Zhang

**Affiliations:** 1 Institute of Molecular Biology and Biotechnology, Bahauddin Zakariya University Multan, Multan, Pakistan; 2 The First Affiliated Hospital of Wenzhou Medical University, Wenzhou, 325000 People’s Republic of China; 3 School of Pharmaceutical Sciences of Wenzhou Medical University, Wenzhou, 325000 People’s Republic of China; 4 Informatics Institute, School of Medicine, The University of Alabama at Birmingham, Birmingham, AL USA; 5 Department of Hepatobiliary Surgery, The First Affiliated Hospital of Wenzhou Medical University, Wenzhou, 325000 People’s Republic of China

## Abstract

**Background:**

Type 2 diabetes mellitus (T2DM) is a worldwide disease that have an impact on individuals of all ages causing micro and macro vascular impairments due to hyperglycemic internal environment. For ultimate treatment to cure T2DM, association of diabetes with immune components provides a strong basis for immunotherapies and vaccines developments that could stimulate the immune cells to minimize the insulin resistance and initiate gluconeogenesis through an insulin independent route.

**Methodology:**

Immunoinformatics based approach was used to design a polyvalent vaccine for T2DM that involved data accession, antigenicity analysis, T-cell epitopes prediction, conservation and proteasomal evaluation, functional annotation, interactomic and in silico binding affinity analysis.

**Results:**

We found the binding affinity of antigenic peptides with major histocompatibility complex (MHC) Class-I molecules for immune activation to control T2DM. We found 13-epitopes of 9 amino acid residues for multiple alleles of MHC class-I bears significant binding affinity. The downstream signaling resulted by T-cell activation is directly regulated by the molecular weight, amino acid properties and affinity of these epitopes. Each epitope has important percentile rank with significant ANN IC_50_ values. These high score potential epitopes were linked using AAY, EAAAK linkers and HBHA adjuvant to generate T-cell polyvalent vaccine with a molecular weight of 35.6 kDa containing 322 amino acids residues. In silico analysis of polyvalent construct showed the significant binding affinity (− 15.34 Kcal/mol) with MHC Class-I. This interaction would help to understand our hypothesis, potential activation of T-cells and stimulatory factor of cytokines and GLUT1 receptors.

**Conclusion:**

Our system-level immunoinformatics approach is suitable for designing potential polyvalent therapeutic vaccine candidates for T2DM by reducing hyperglycemia and enhancing metabolic activities through the immune system.

## Background

Type 2 diabetes mellitus (T2DM), a non-insulin dependent metabolic disorder, is a pandemic disease affecting large set population of the world [1]. It is responsible for 90% of total diabetic population and sixth prime cause of disability. T2DM is characterized by the inability of pancreatic β-cells to produce enough insulin resulting hyperglycemia and the inability of insulin to bind with its receptors restrict the absorption of glucose (insulin resistance) into the cells [2, 3]. Disease prevalence is increasing for sure due to unknown causes and the lack of therapeutic strategies [4, 5].

Normally, insulin regulates the absorption of glucose through glucose transporter type 4 (GLUT-4) protein channels [6] present in cell membranes. In case of insulin impairment, the absorption of glucose by GLUT-4 doesn’t occur, causing T2DM [7]. The glucose transporters (GLUT1 and GLUT4) facilitate glucose transport into cells. GLUT1 is insulin-independent and is widely distributed in different tissues [8, 9]. Cells need growth factors to facilitate glucose absorption for subsistence and development. T-cell stimulation leads to fast proliferation and differentiation into effector cells that release cytokines and mediate the immunity [10–12].

The non-insulin growth factors such as cytokines including interleukin IL3 and IL7 may absorb the glucose through glucose transporter type 1 (GLUT-1) proteins. These cytokines can trigger the cascade of important signals to promote glucose uptake via different pathways. GLUT1 proteins in response to cytokines released by active immune components facilitate the constitutive, insulin-independent glucose uptake in most of the cells including hematopoietic and muscle cells [13–15].

The progression of T2DM is also associated to abnormal immune responses [16], and therefore the cytokine-mediated regulation of GLUT-1 can be thought of playing some significant role in this respect. The metabolic reprogramming is shaped to help definite cell functions [17] and glucose uptake delivers a key metabolic control point through the GLUT family of facilitative glucose transporters.

**Fig. 1:**
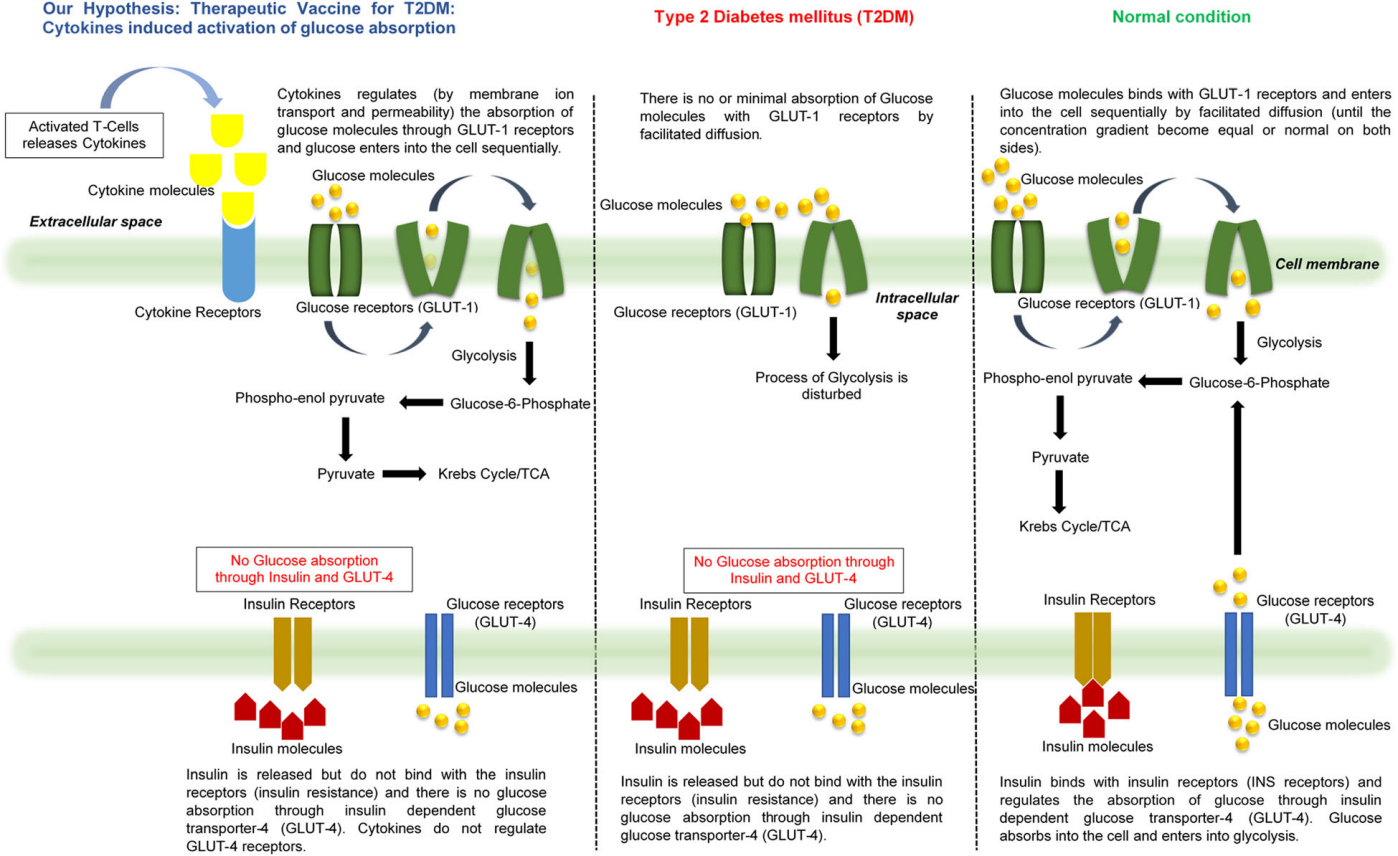
Our hypothesis would facilitate the glucose absorption through cytokines production and GLUT-1 channels to manage insulin resistance

In this study, we hypothesize the development of potential immunotherapeutic vaccine candidates for the activation and secretion of cytokines (IL-1, IL-3, and IL-7) to facilitate glucose absorption and cure T2DM. The issues related to insulin resistance could be minimized through alternative non-insulin dependent GLUT1 pathway. We predicted the T-cell epitopes and analyzed the *in-silico* binding affinity with MHC class-I molecules. Our vaccine would target the T-cells resulting the secretion of interleukins. Instead of insulin dependent GLUT-4 channels, these interleukins open up the GLUT-1 proteins and regulate the glucose absorption. Our hypothesis has been illustrated in Fig. 1. This study would modulate the therapeutic strategies to manage type 2 diabetes mellitus.

## Method

### Retrieval of protein data

**Fig. 2:**
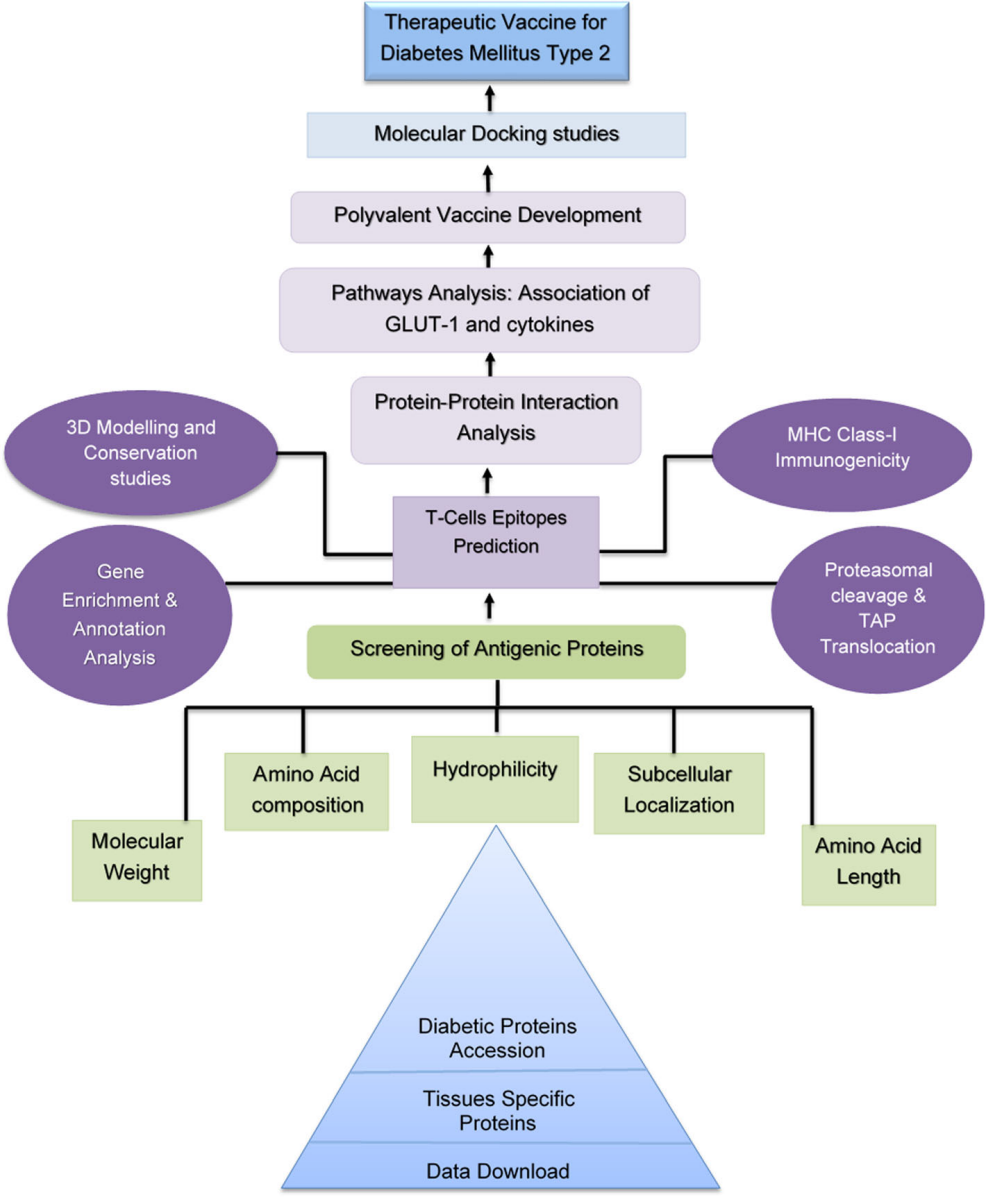
Our integrated framework to design polyvalent vaccine for T2DM by immunoinformatics approach

The tissue specific (bearing GLUT-1 receptors) protein sequences were retrieved from NCBI and Uniprot databases (Supplementary Table 1). The data covered all information including protein names, gene symbols, Uniprot accession numbers, protein description and sequences. The currently available proteins sequences associated to type 2 diabetes mellitus (Supplementary Table 2) were accessed from diabetic databases. We carried out this study using integrated framework (Fig. 2) by computational tools, databases, online servers and software (Table 1).

**Table 1:** Tools, Databases and Software used in this study

Database/Tools	Web Link	Purpose	References
NCBI	https://www.ncbi.nlm.nih.gov/	Accession of Data	–
T2D@ZJU	http://pharminfo.zju.edu.cn/t2d	Diabetes associated genes retrieval	[18]
DAPD	http://mkarthikeyan.bioinfoau.org/dapd/	Retrieval of proteins	[19]
ToxinPred & PredSTP	https://webs.iiitd.edu.in/raghava/toxinpred/index.html	Prediction of toxic peptides	[20, 21]
UniProt	http://www.uniprot.org	Screening of Diabetic proteins	[22]
Compare Two Lists	http://jura.wi.mit.edu/bioc/tools/compare.php	Comparison	[23].
SPpred	http://crdd.osdd.net:8081/sppred/submit.jsp	Solubility/hydrophilicity determination	–
PROTPARAM	http://web.expasy.org/protparam/	Amino Acid composition	[24]
CELLO	http://cello.life.nctu.edu.tw/	Subcellular Localization Prediction	[25]
PROPRED I	http://crdd.osdd.net/raghava/propred1/	Prediction of T-Cell epitopes	[24]
HADDOCK 2.2	https://haddock.science.uu.nl/	Docking of epitopes	[26]
IEDB (NetChop)	http://tools.iedb.org/netchop/result/	Proteasomal cleavage prediction	[27].
IEDB	http://tools.iedb.org/conservancy/	Epitope Conservancy Analysis	[28]
PEPFOLD	http://bioserv.rpbs.univ-paris-diderot.fr/services/PEP-FOLD/	3D Modelling of Epitopes	[29]
DAVID Tool	https://david.ncifcrf.gov/home.jsp	Functional Annotation	[30]
HAPPI	http://discovery.informatics.uab.edu/HAPPI/	Interactomic analysis	[31]
STRING	https://string-db.org/	Interactomic analysis	[32].
Cytoscape	http://www.cytoscape.org/	Protein-protein interaction	[33]
MOE	https://www.chemcomp.com/	Epitopes binding energy	–
ITASSER	https://zhanglab.ccmb.med.umich.edu/I-TASSER/	3D Model generation	[34–36]
Chimera	https://www.cgl.ucsf.edu/chimera/	Visualization of proteins	[37]
FUNRICH	http://www.funrich.org/	Gene enrichment analysis	[38]
ERRAT	http://servicesn.mbi.ucla.edu/ERRAT/	Error in model estimation	[39]
QMean	https://swissmodel.expasy.org/qmean/	Quality of model	[40, 41]
Rampage Analysis	http://mordred.bioc.cam.ac.uk/~rapper/rampage.php	Protein Quality	[42]
3D Refine	http://sysbio.rnet.missouri.edu/3Drefine/	Refinement of polyvalent model	[43–45]
Antigen Pro	http://scratch.proteomics.ics.uci.edu/	Antigenicity	[46]
Vaxijen	http://www.ddg-pharmfac.net/vaxijen/VaxiJen/	Antigenicity	[47–49]
AlgPred	http://crdd.osdd.net/raghava/algpred/submission.html	Allergenicity	–
Sol Pro	http://scratch.proteomics.ics.uci.edu/explanation.html#SOLpro	Solubility of proteins	[46]
Expasy	http://web.expasy.org/compute_pi/	Molecular Weight prediction	[24]

### Screening of antigenic proteins

We mapped the non-tissue specific list of diabetic proteins with the entire list of tissue specific proteins using “Compare Two Lists” tool [23] to shortlist the T2DM associated proteins in the relevant tissues. The antigenicity of these shortlisted proteins were determined (based on the significant threshold level > 0.5) using “VaxiJen v2.0” server [47]. To select the robust and effective antigenic proteins, these proteins were further screened and filtered based on the molecular weight (threshold of > 85 kDa) using Expasy Compute pI/MW tool [24, 50], hydrophilicity using SPpred server, amino acid composition using Expasy ProtParam tool [24] and subcellular localization using CELLO v.2.5: Subcellular Localization Predictor tool [25].

### T-cell epitopes prediction and immunogenicity analysis

We predicted multi-allelic T-Cells epitopes of selected proteins using ProPred-I online server [24]. This server identified the MHC Class-I regions in the input sequences of selected proteins by applying matrices of all 47 alleles of this class. These epitopes have the potential to target several alleles of MHC Class-I. The multi-allelic epitopes-based vaccine has more worth and chance of success as compared to the vaccine that target only one type of allele in the whole population. The proteasomal cleavage sites of these antigenic proteins were predicted using NetChop predictor of IEDB tool [27]. The proteasomal prediction is important to find out the potential immunogenic regions in the selected proteins. We verified the immunogenicity of MHC Class-I epitopes using IEDB server. This tool uses amino acid properties and their position within the peptide to find the immunogenicity of a peptide MHC (pMHC) complex [27].

### Conservancy analysis and physicochemical properties prediction

The conservational analysis of selected epitopes was analyzed using IEDB server [27] to observe the conservancy between epitopes and the proteins. We predicted the physicochemical properties of candidate peptides including half-life, instability index, net charge, peptide toxicity, and hydropathicity using ToxinPred and PredSTP servers [20, 21]. The half-life of peptide varies among species and it is important to determine the half-life of peptides in humans. The stability profile of peptide is indicated by the value of the instability index. The charge of a peptide is pH-dependent. At the isoelectric point, the net charge on protein is zero. The solubility of protein is lowest at its isoelectric point. The hydropathicity analysis provides detail information about individual amino acid that enables us to predict the overall three-dimensional structure of a protein from its amino acid sequence. The Support Vector Machine (SVM) model of amino acid composition was used to predict the toxicity profile of peptides.

### Modelling of epitopes

The three-dimensional (3D) structure of peptides is usually helpful to understand its topological description, biological activity and function. The protein structure prediction often offers a suitable alternative to facilitate structure-based studies. 3D models of selected epitopes were generated by using PEP-FOLD [29], I-Tasser [34] and Chimera [37] tools to observe epitopic regions and underlying pattern of amino acids. These tools require amino acid input sequences to build the peptide folds.

### Gene enrichment and annotation analysis

Gene enrichment and annotation analysis of these peptides were performed using FunRich [38] and David tool [30]. The annotation profiles including functional and cluster details were studied.

### Protein-protein interaction (PPI) analysis

We studied PPI network of selected antigenic proteins to carryout system level investigations. The target interactions of selected source proteins were retrieved using STRING database [32]. This database provides the comprehensive detail about the interactions, functions and pathways of sample proteins. The PPI network was constructed by using Cytoscape v3.6.0 software [33].

### Pathways analysis

To study the physiological role of these potential vaccinating proteins, we designed and constructed the integrated, interactive and metabolic network of T2DM-related proteins and observed the correlation between these pathways. Cellular and signaling pathways were reconstructed from the combined gene signatures using PathVisio3 tool. These proteins were mapped and curated using KEGG (Kyoto Encyclopedia of Genes and Genomes) and WIKI pathways on the basis of literature and database evidence.

### Polyvalent vaccine assembly

Polyvalent vaccine was designed and constructed by linking selected epitopes of MHC Class-I using potential linkers. We used AAY and EAAAK linkers to link 13-epitopes to minimize the undesirable attachment of their ends that might cause change in amino acid arrangement and even the functionality of proteins [51]. To enhance the immunogenicity, the amino acid sequence (159 residues) of the heparin-binding hemagglutinin (HBHA) was used as adjuvant. On both ends of HBHA, EAAAK linkers were used to make it non-reactive [51]. Epitopes cannot be bluntly linked with each other to design a polyvalent vaccine. The arrangement of amino acid sequences is critical and their order is based on their affinity and compatibility with each other. To design the best and compatible construct of selected epitopes, we used the HADDOCK 2.2 server [26] to analyze the binding affinity of these epitopes. Initially, the binding affinity of each epitope was evaluated followed by the combined sequences to shortlist the best construct. The refinement and residues determination were carried out using HADDOCK: Refinement Interface and Cport modules [26] respectively.

### Modelling of polyvalent vaccine and quality estimation

The polyvalent vaccine construct was modelled using I-Tasser server [34]. The input FASTA format of amino acid sequence was used to generate 3D model. The model was refined using 3D Refine tool. The quality of the model was estimated by Ramachandran plot and quality model energy analysis (QMEAN) score. Ramachandran plot accurately describes the protein conformation and illustrates the favorable regions for backbone dihedral angles against amino acid residues in protein structure. Similarly, QMean score describes the protein quality on the basis of different geometrical aspects of its structure.

### *In-Silico* binding affinity analysis

The binding affinity of polyvalent vaccine model with MHC class-I molecule was analyzed using Molecular Operating Environment (MOE) software. The MOE software was set at default parameters and the interaction between molecules was visualized. The active binding sites of the MHC molecule was observed and the binding interaction between the amino acid residues was assessed based on binding energy.

## Results

### Identifying antigenic proteins

**Fig. 3:**
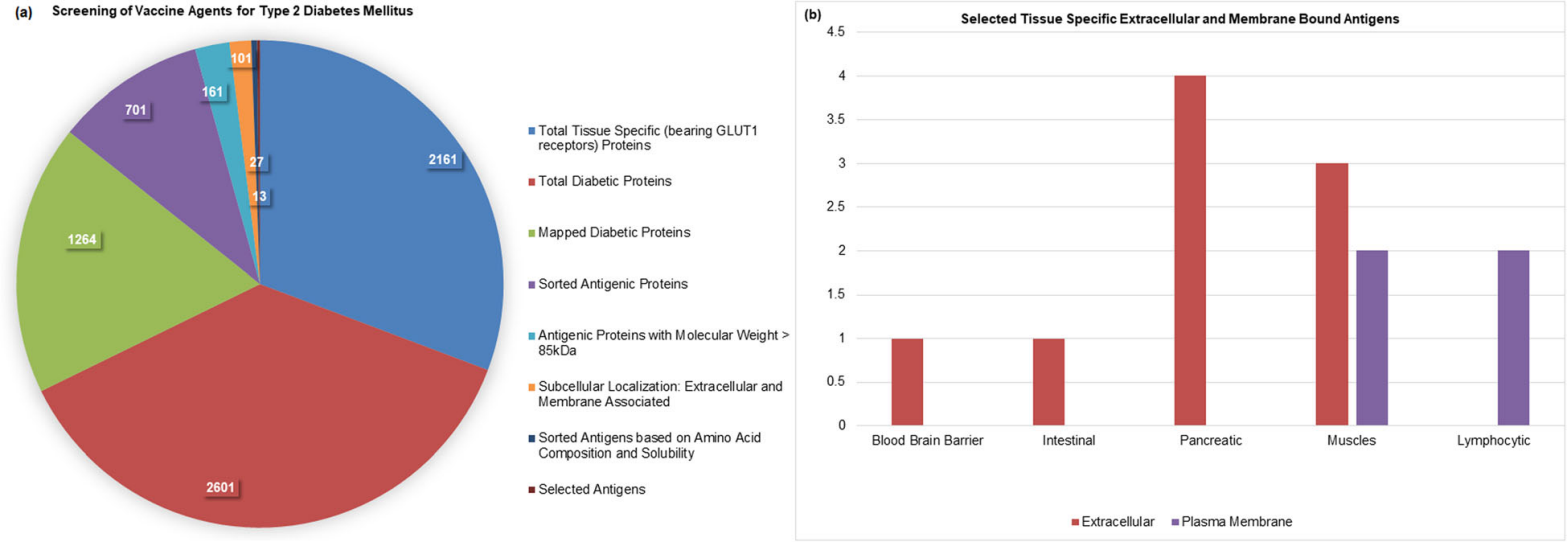
**a** Screening of vaccine agents for T2DM using synchronized steps **b** Distribution of selected extracellularly and membrane bound antigenic proteins in different tissues

From the list of 2601 diabetic proteins, we identified 13-antigenic tissue specific proteins associated with T2DM based on antigenicity, molecular weight, subcellular localization, amino acid composition, length of protein sequence and solubility using successive screening tools (Fig. 3a). These selected extracellular and membrane bound proteins are associated to blood brain barrier, pancreas, muscles, lymphocytes and intestines (Fig. 3b). These proteins like interleukin-32 (IL-32), insulin like growth factor 1 (IGF1), transforming growth factor beta-1 (TGFB1), toll like receptors-3 (TLR3) and ras-related C3 botulinum toxin substrate 1 (RAC1), with their important biological functions including immune modulation, insulin signaling, cell survival, immune components activation and phosphorylation during glucose metabolism (Table 2).

**Table 2:** List of 13-Potential Antigenic Proteins associated with Type 2 Diabetes Mellitus

Tissue Name	Gene Symbol	Uniprot_ID	Protein Names	Subcellular Localization
Blood Brain Barrier	RAC1	RAC1_HUMAN	Ras-related C3 botulinum toxin substrate 1 (Rho family)	Extracellular
Intestinal	MMP2	MMP2_HUMAN	Matrix metallopeptidase 2 (Gelatinase A, 72 kDa gelatinase)	Extracellular
Pancreatic	TLR3	TLR3_HUMAN	Toll-like receptor 3 (CD antigen CD283)	Extracellular
Pancreatic	ITGB1	ITB1_HUMAN	Integrin beta-1 (Fibronectin receptor subunit beta) (Glycoprotein IIa)	Extracellular
Pancreatic	LTF	TRFL_HUMAN	Lactotransferrin (Lactoferrin) (Growth-inhibiting protein 12)	Extracellular
Pancreatic	IL32	IL32_HUMAN	Interleukin-32 (IL-32) (Natural killer cells protein 4) (Tumor necrosis factor alpha-inducing factor)	Extracellular
Muscles	LRP6	LRP6_HUMAN	Low-density lipoprotein receptor-related protein 6 (LRP-6)	Plasma membrane
Muscles	LEPR	LEPR_HUMAN	LEPR protein (Fragment)	Extracellular
Muscles	TGFB1	TGFB1_HUMAN	Transforming growth factor, beta 1 (Camurati-Engelmann disease)	Extracellular
Muscles	IGF1	IGF1_HUMAN	Insulin-like growth factor I (IGF-I) (Somatomedin-C)	Extracellular
Muscles	HLA-DRA	DRA_HUMAN	HLA-DRA (MHC class II antigen) (major histocompatibility complex	Plasma Membrane
Lymphocytic	ABCB1	A1L471_HUMAN	ATP-binding cassette, (MDR/TAP), member 1	Plasma Membrane
Lymphocytic	TRPM7	TRPM7_HUMAN	Transient receptor potential cation channel subfamily M member 7	Plasma Membrane

### MHC peptide binding and prediction of T-cell epitopes

T-cell epitopes (9-mers in length) of 13-selected proteins were predicted. These MHC class-I specific epitopes are multi-allelic in nature and could target several alleles of MHC Class-I of human population. We determined 13-immunogenic T-cell epitopes with significant percentile rank (% rank: 0.1), MHC Class-I immunogenicity score (> 0.5) and multiple alleles hit. These selected T-cell epitopes of 13-proteins as potential candidate antigens have been listed in Table 3.

**Table 3:** Predicted T-Cell Epitopes (antigenic and immunogenic) of selected proteins

UNIPROT_ID	Gene Symbol	Molecular Mass (KDa)	T-Cell Epitopes	Peptide Position	No. of Alleles	Antigenicity	MHC Class-I Immunogenicity
RAC1_HUMAN	RAC1	23	FDEAIRAVL	188	10	0.567	0.30733
MMP2_HUMAN	MMP2	74	LVATFWPEL	508	10	0.6217	0.42341
TLR3_HUMAN	TLR3	104	GCFHAIGRL	215	14	0.528	0.29277
ITB1_HUMAN	ITGB1	88	TGPDIIPIV	725	7	0.9421	0.342
TRFL_HUMAN	LTF	78	GYTGAFRCL	546	7	1.2559	0.20718
IL32_HUMAN	IL32	27	LQTWWHGVL	165	10	1.0906	0.53436
LRP6_HUMAN	LRP6	180	LDQPRAIAL	137	10	1.6709	0.17365
LEPR_HUMAN	LEPR	75	MWIRINHSL	511	10	0.6983	0.14943
TGFB1_HUMAN	TGFB1	44	LYIDFRKDL	298	10	0.6744	0.0592
IGF1_HUMAN	IGF1	22	QKEGTEASL	161	2	1.1216	0.13501
DRA_HUMAN	HLA-DRA	29	NVPPEVTVL	109	11	0.5111	0.17848
A1L471_HUMAN	ABCB1	141	LLERFYDPL	1083	13	1.3167	0.20734
TRPM7_HUMAN	TRPM7	213	KQTEEGGNL	330	10	1.6003	0.26757

### Proteasomal cleavage and epitope conservation analysis

**Fig. 4:**
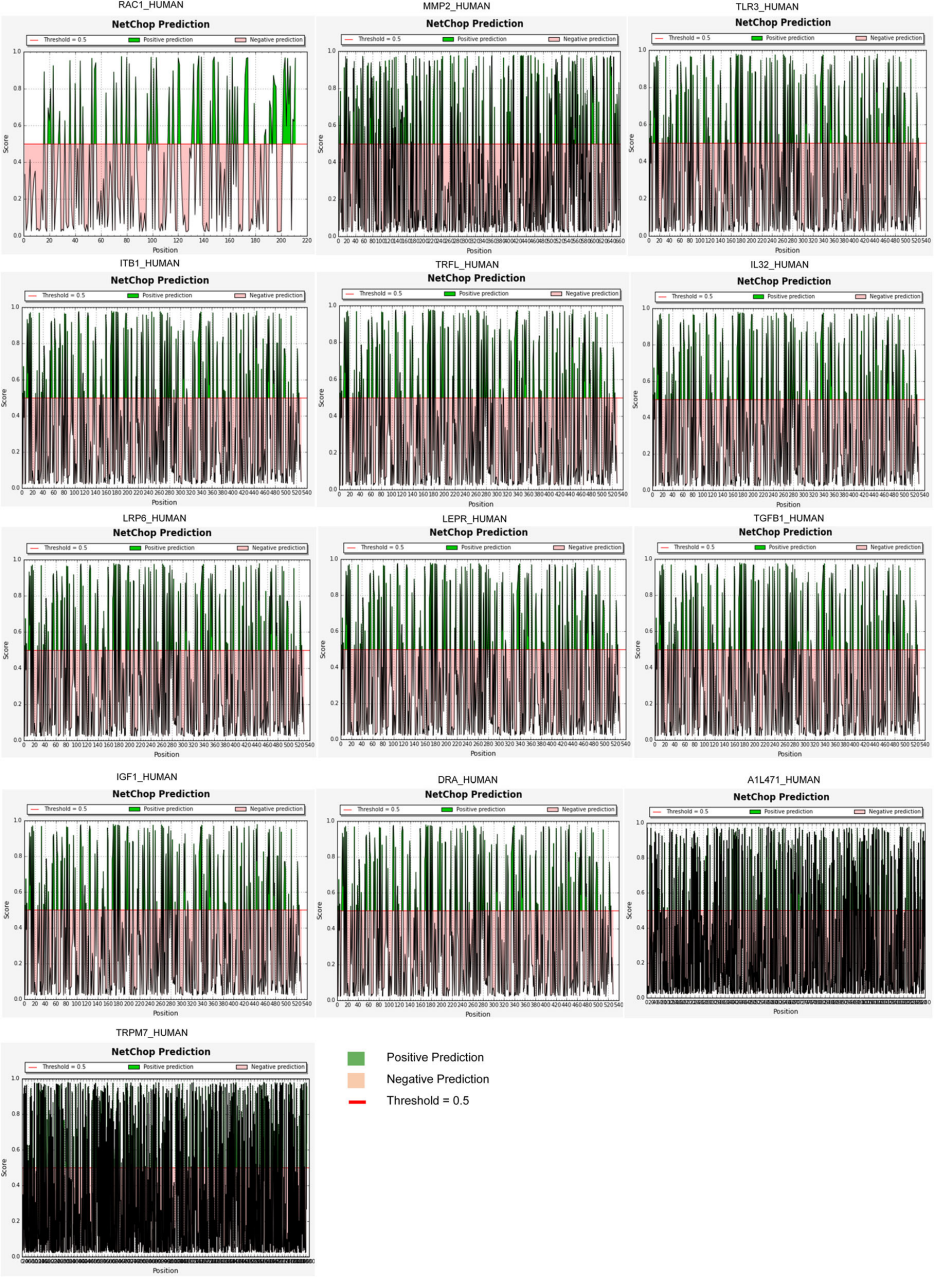
Proteasomal and TAP translocation prediction of the selected antigenic proteins by NetChop platform of Immune Epitope Database (IEDB). Green and pink colors indicate the positive and negative prediction at threshold of 0.5

We applied NetChop tool to predict proteasomal cleavage that depends upon a neural network. This method identified the C-terminal at cleavage sites with the threshold value of 0.5 to categorize the cleavage and non-cleavage sites. We observed the cleavage (positive predictions) and non-cleavage (negative predictions) sites of antigenic proteins by proteasomes indicating their significant role in antigen presentation to MHC class-I molecule (Fig. 4). The scores of combined predictions of proteasomal cleavage, TAP translocations, and MHC binding shows each peptide intrinsic capacity of being a T-cell epitope.

Epitope conservation analysis of antigenic peptides showed broad coverage and 100% protein sequence matches. Conservation analysis was performed by IEDB and epitopes were designed by ProPred-I identified the MHC Class-I regions in the input sequences of selected proteins by applying matrices of all 47 alleles of this class. Those epitopes were selected that are present in maximum alleles out of 47 reported alleles of MHC class I. These epitopes have the potential to target several alleles of MHC Class-I. The multi-allelic epitopes-based vaccine has more worth and the chance of success as compared to the vaccine that targets only one type of allele in the whole population. The conserved epitopes have significant ability to provide the effective immune response (Table 4). Most of the predicted epitopes have instability index < 40 indicating their stability. A good epitope must be stable and stay in the body to activate the immune components. Most of our predicted peptide has a half-life of more than 5 h and all are non-toxic. SVM score is used to classify either the epitope is toxic or non-toxic based on the threshold value (non-toxic: < 5). Almost all predicted epitopes have an SVM score of less than 5 indicating non-toxicity. Amino acid chains are either hydrophobic or hydrophilic. The lesser value of hydropathicity means the more hydrophilic character of epitopes (Table 5).

**Table 4:** T-Cell Epitopes conservation analysis

Epitope #	Epitope Name	Epitope Sequence	Epitope Length	Sequence Similarity <= 100%	Minimum Identity	Maximum Identity
1	ws-separated-0	FDEAIRAVL	9	100.00% (1/1)	100.00%	100.00%
2	ws-separated-1	LVATFWPEL	9	100.00% (1/1)	100.00%	100.00%
3	ws-separated-2	GCFHAIGRL	9	100.00% (1/1)	100.00%	100.00%
4	ws-separated-3	TGPDIIPIV	9	100.00% (1/1)	100.00%	100.00%
5	ws-separated-4	GYTGAFRCL	9	100.00% (1/1)	100.00%	100.00%
6	ws-separated-5	LQTWWHGVL	9	100.00% (1/1)	100.00%	100.00%
7	ws-separated-6	LDQPRAIAL	9	100.00% (1/1)	100.00%	100.00%
8	ws-separated-7	MWIRINHSL	9	100.00% (1/1)	100.00%	100.00%
9	ws-separated-8	LYIDFRKDL	9	100.00% (1/1)	100.00%	100.00%
10	ws-separated-9	QKEGTEASL	9	100.00% (1/1)	100.00%	100.00%
11	ws-separated-10	NVPPEVTVL	9	100.00% (1/1)	100.00%	100.00%
12	ws-separated-11	LLERFYDPL	9	100.00% (1/1)	100.00%	100.00%
13	ws-separated-12	KQTEEGGNL	9	100.00% (1/1)	100.00%	100.00%

**Table 5:** Physicochemical properties of Predicted T-Cell epitopes

Uniprot ID	Peptide Sequence	SVM Score	Prediction	Hydropathicity	Charge	Half-Life (Hours)	Instability Index
RAC1_HUMAN	FDEAIRAVL	−0.87	Non-Toxin	0.82	−1	1	22.6
MMP2_HUMAN	LVATFWPEL	−1.22	Non-Toxin	1.08	−1	5.5	41.91
TLR3_HUMAN	GCFHAIGRL	−0.4	Non-Toxin	0.77	1.5	30	8.89
ITB1_HUMAN	TGPDIIPIV	−0.52	Non-Toxin	1.1	−1	7.2	−21.56
TRFL_HUMAN	GYTGAFRCL	−0.62	Non-Toxin	0.4	1	30	−7.44
IL32_HUMAN	LQTWWHGVL	−1.31	Non-Toxin	0.24	0.5	5.5	43.2
LRP6_HUMAN	LDQPRAIAL	−1.32	Non-Toxin	0.29	0	5.5	21.91
LEPR_HUMAN	MWIRINHSL	−0.55	Non-Toxin	0.2	1.5	30	8.89
TGFB1_HUMAN	LYIDFRKDL	−1.01	Non-Toxin	−0.2	0	5.5	0.51
IGF1_HUMAN	QKEGTEASL	−0.81	Non-Toxin	−1.19	−1	0.8	20.86
DRA_HUMAN	NVPPEVTVL	−1.14	Non-Toxin	0.61	−1	1.4	61.57
A1L471_HUMAN	LLERFYDPL	−1.17	Non-Toxin	−0.02	−1	5.5	71.42
TRPM7_HUMAN	KQTEEGGNL	−0.84	Non-Toxin	−1.73	− 1	1.3	97.1

### Molecular modeling of epitopes

**Fig. 5:**
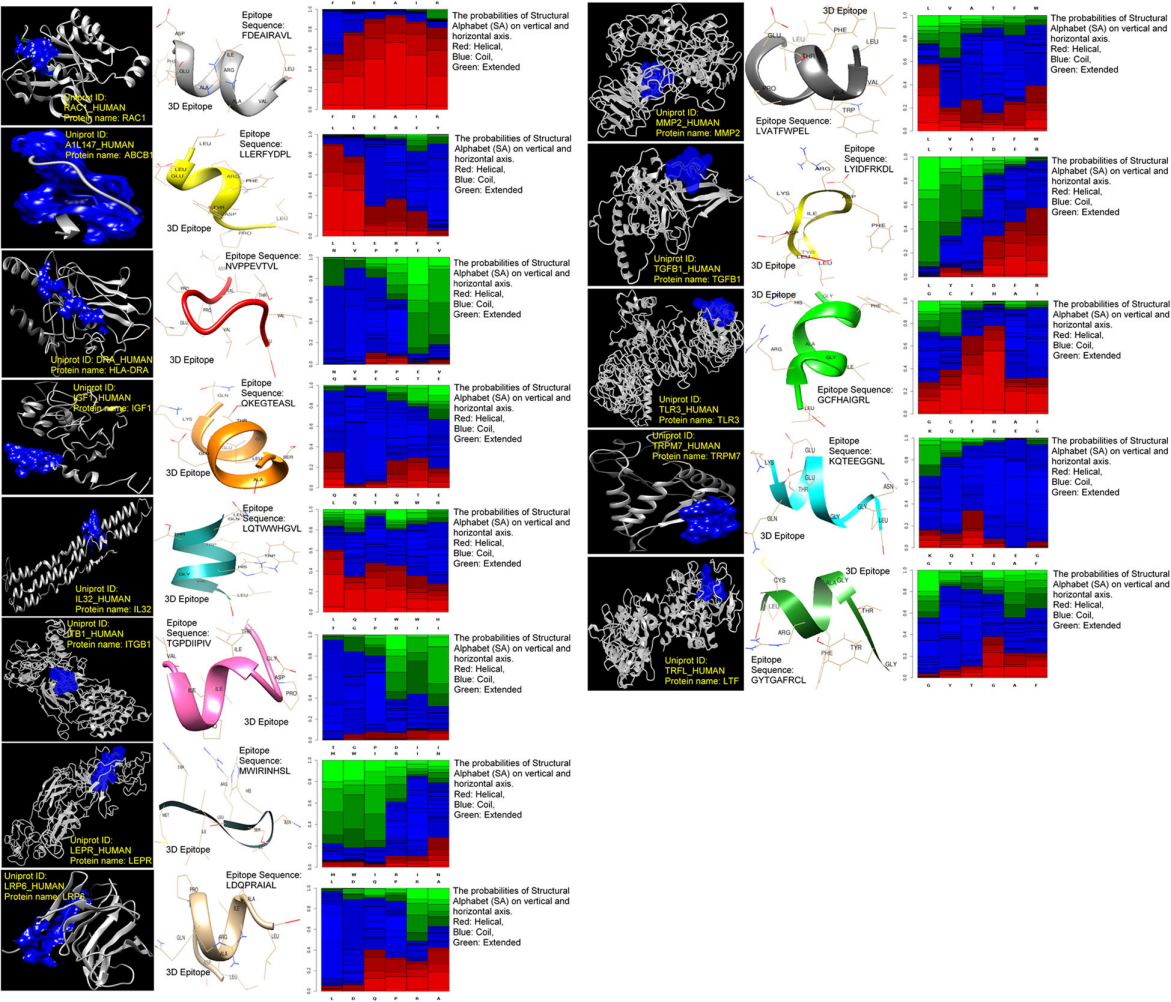
Generation of 3D models of selected epitopes using I PEPFOLD and Chimera tools. Heats maps designed by PEPFOLD show probabilities of structural alphabet (SA) on horizontal and vertical axis. Red: Helical, Blue: Coil and Green: Extended

We predicted the 3D models of 13-epitopes from input 9-mers amino acid sequences using PEP-FOLD server. In local structure analysis, the probabilities of each Structural Alphabet (SA) on vertical and horizontal axis has been shown (Fig. 5). In heat map Figure, red color codes indicate the helical form of the structure, green presents extended and blue indicates coil conformations. This server found lowest energy conformations with an average RMSD of 2.1 Å.

### Functional annotation and gene enrichment analysis

**Fig. 6:**
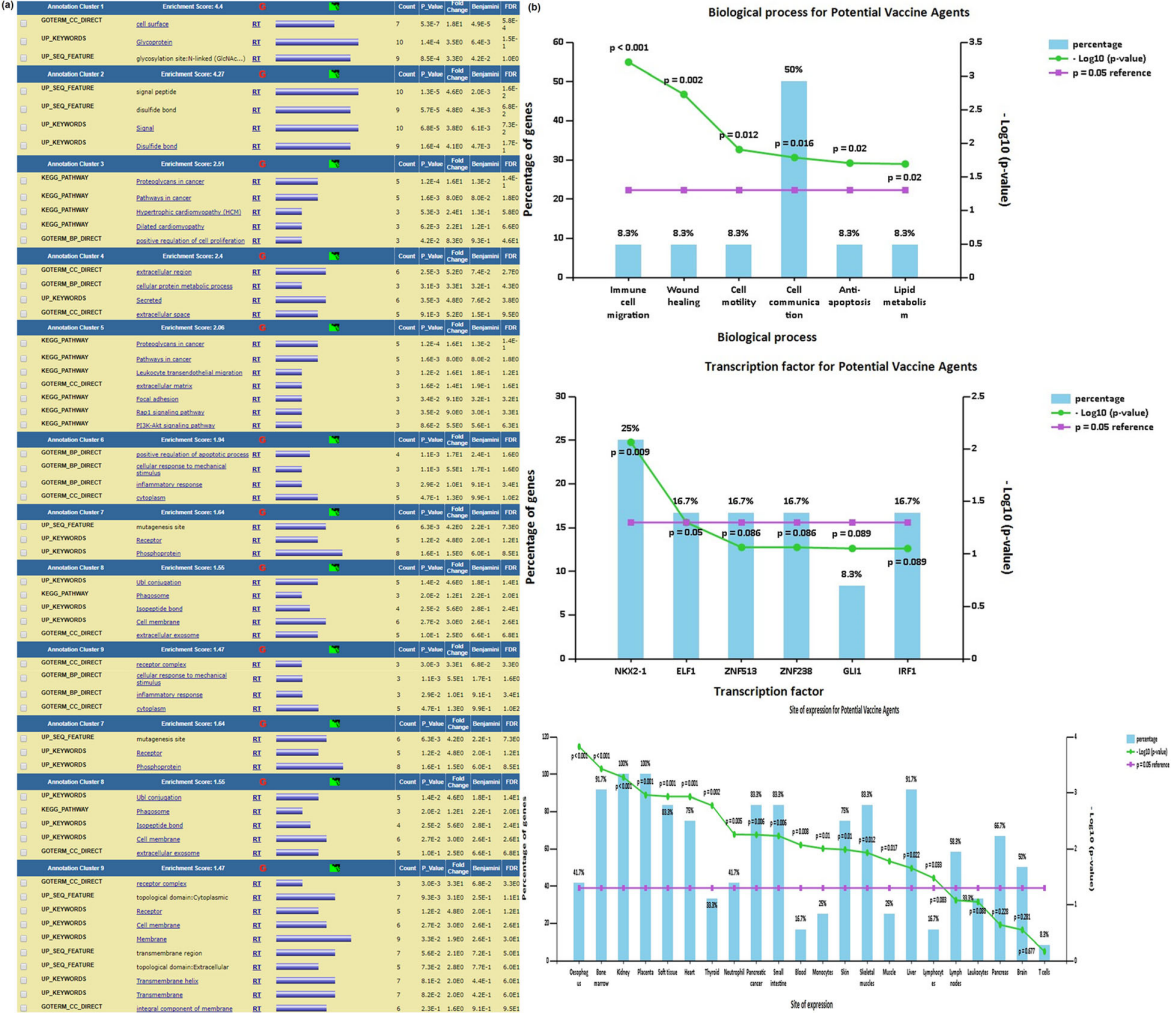
**a** Annotation clusters identifies the enriched proteins that play an important role in immune signaling and metabolic pathways involved in regulation of glucose absorption. **b** Functional annotation categorizes proteins biological functions, transcription factor of T2DM associated proteins with significant *p*-values (< 0.05) and site of expression of these proteins in different tissues

The functional annotation and enrichment of the T2DM associated proteins were analyzed using DAVID and FunRich tools. The functional annotation identifies protein functional domains, disease associations, protein-protein interactions and biological pathways. This cluster analysis showed that these proteins are significantly associated with cell-cell communication, glycosylation, cell surface responses and cell proliferation regulation (Fig. 6a). The biological role of these potential antigenic proteins has been importantly seen in immune cell migration, wound healing, cell mobility, cell communication, anti-apoptosis and lipid metabolism. Their transcription factors involve NKX2–1, ELF1. ZNF513, ZNF238, GLI1 and IRF1 with significant *p*-values (*p* < 0.05) expressed in different tissues (Fig. 6b).

### Protein-protein interaction analysis

**Fig. 7:**
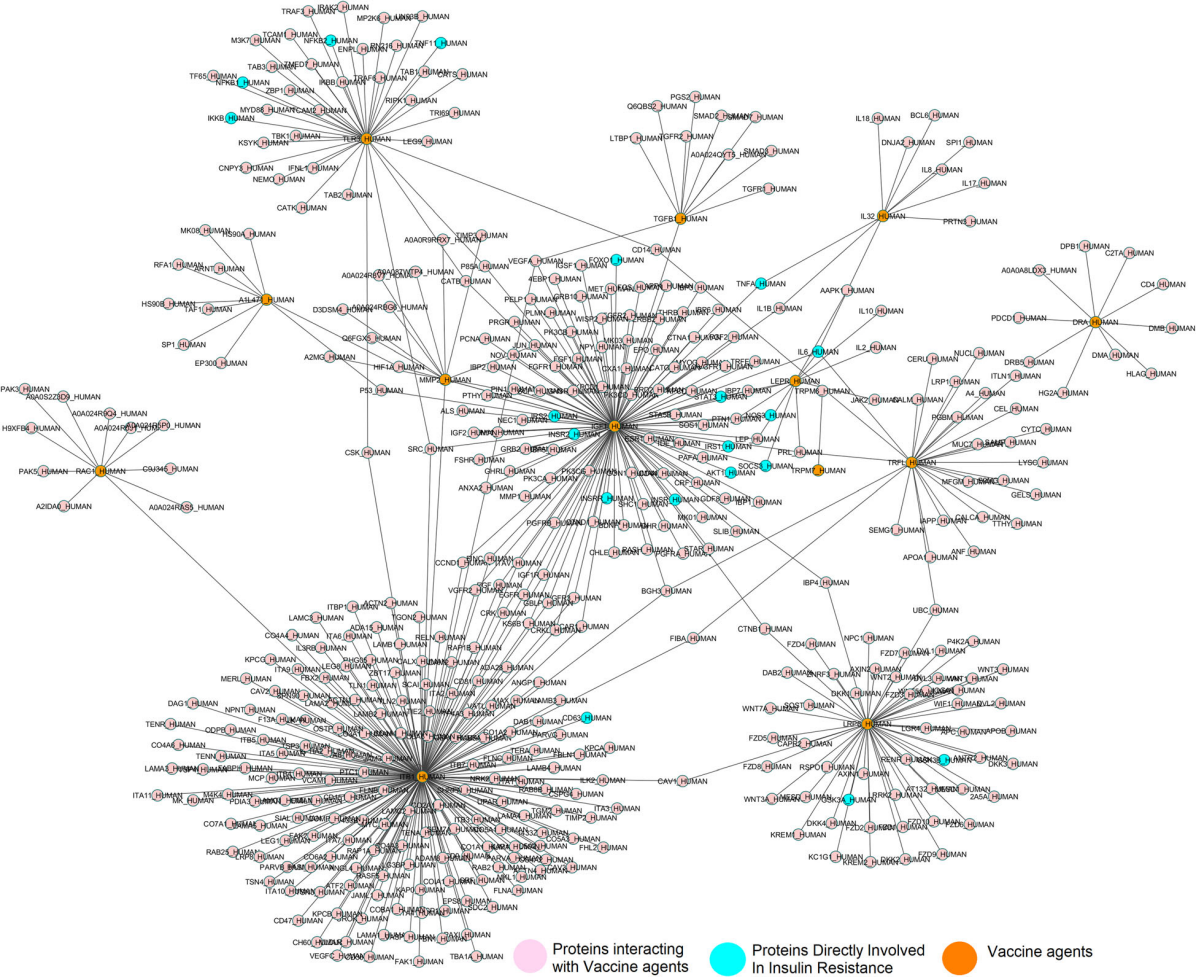
Interactomic analysis and construction of PPI network using Cytoscape software. Protein–protein interaction network of selected antigenic proteins. Nodes and edges (lines) denote proteins and their interactions respectively. Network comprises: Orange nodes for vaccine candidates; turquoise nodes represent T2DM and insulin resistance associated proteins while the pink nodes are other functional proteins. Network contains 448 nodes and 484 edges using the high-confidence data

To understand and analyze the topology and functional annotation of protein-protein interaction (PPI) of T2DM-antigenic proteins, the PPI network was constructed. The entire network contained high scoring interaction partners (confidence score: > 0.9). The main component of this network contained 448 nodes and 484 edges (nodes represent proteins and edges represent interaction). This interaction network was largely segregated into three neighborhoods: orange nodes indicate the potential vaccine candidate proteins; turquoise nodes represent the proteins that are directly involved in insulin resistance while the pink nodes are other functional proteins. The topological analysis revealed the direct interaction of these antigenic proteins with the target proteins involved in apoptosis, aging, cell division, metabolism, glucose transportation, transcriptional factors activation and T-cell stimulation. Of these proteins, IGF1 (IGF1_HUMAN) is principally interacting with INSR (facilitates the action of insulin), FOXO1 (metabolic regulation under stress conditions), STAT3 (involved in signal transduction and cellular reactions to interleukins) PK3CA (initiates cascades of cell growth, motility, survival and proliferation pathways), AKT1 (regulates cell cycle, angiogenesis and metabolic processes), TNFA (anti-tumor activities and cell differentiation), SOCS3 (negatively regulates cytokine signaling) and IL-6 (immune components differentiation and critical immune responses). In the same way, TLR3 (TLR3_HUMAN) is interrelating with IKKB (controls the production of immune mediators and provides security against apoptosis), NFKB1 (final product of many signal transduction pathways stimulated by inflammation, apoptosis and cell growth) and LRP6 (LRP6_HUMAN) interacts with GSK3B (regulator of glucose homeostasis) (Fig. 7). Such interaction of antigenic proteins makes them effective to regulate the immune responses and metabolic pathways.

### Pathways analysis

**Fig. 8:**
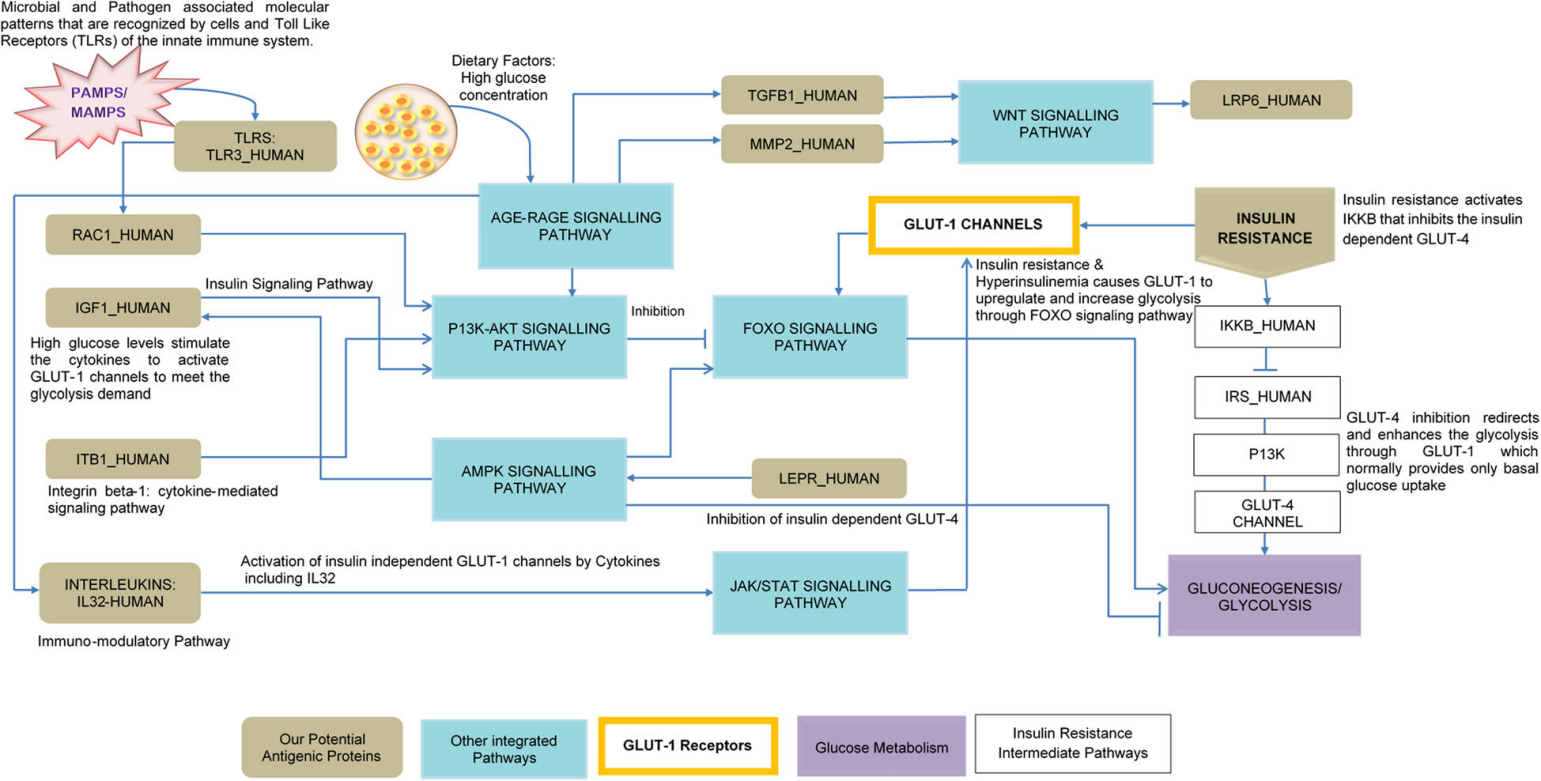
Pathway analysis indicates the association of our selected antigenic proteins with T2DM related vital pathways, immune signaling system and GLUT-1 receptors

The pathways modeling showed that several pathways are involved in T2DM- pathophysiology and they are connected with our core vaccinating proteins. This analysis indicated the association of antigenic protein with immune system and cellular signaling mechanism. Besides insulin-signaling and insulin-resistance pathways, the others are AMPK signaling, JAK/STAT pathway, FOXO signaling, P13K-AKT signaling, and WNT pathways are associated to our potential antigenic proteins and responsible for the activation of immune components and GLUT-1 receptors to regulate T2DM. It was found that some proteins like RAC1_Human, IGF1_Human, ITB1_Human were associated with the activation of P13K-AKT pathway which is the main regulator of many cellular processes like cell growth, survival and glucose metabolism. In insulin resistance, the FOXO signaling pathway activates GLUT-1 and inhibition of GLUT-4 by IKKB. The immune-regulatory mechanism and increased production of cytokines is associated with the activation of JAK/STAT pathway and GLUT-1 receptors to control glucose metabolism and T2DM (Fig. 8).

### Polyvalent vaccine construct

**Fig. 9:**
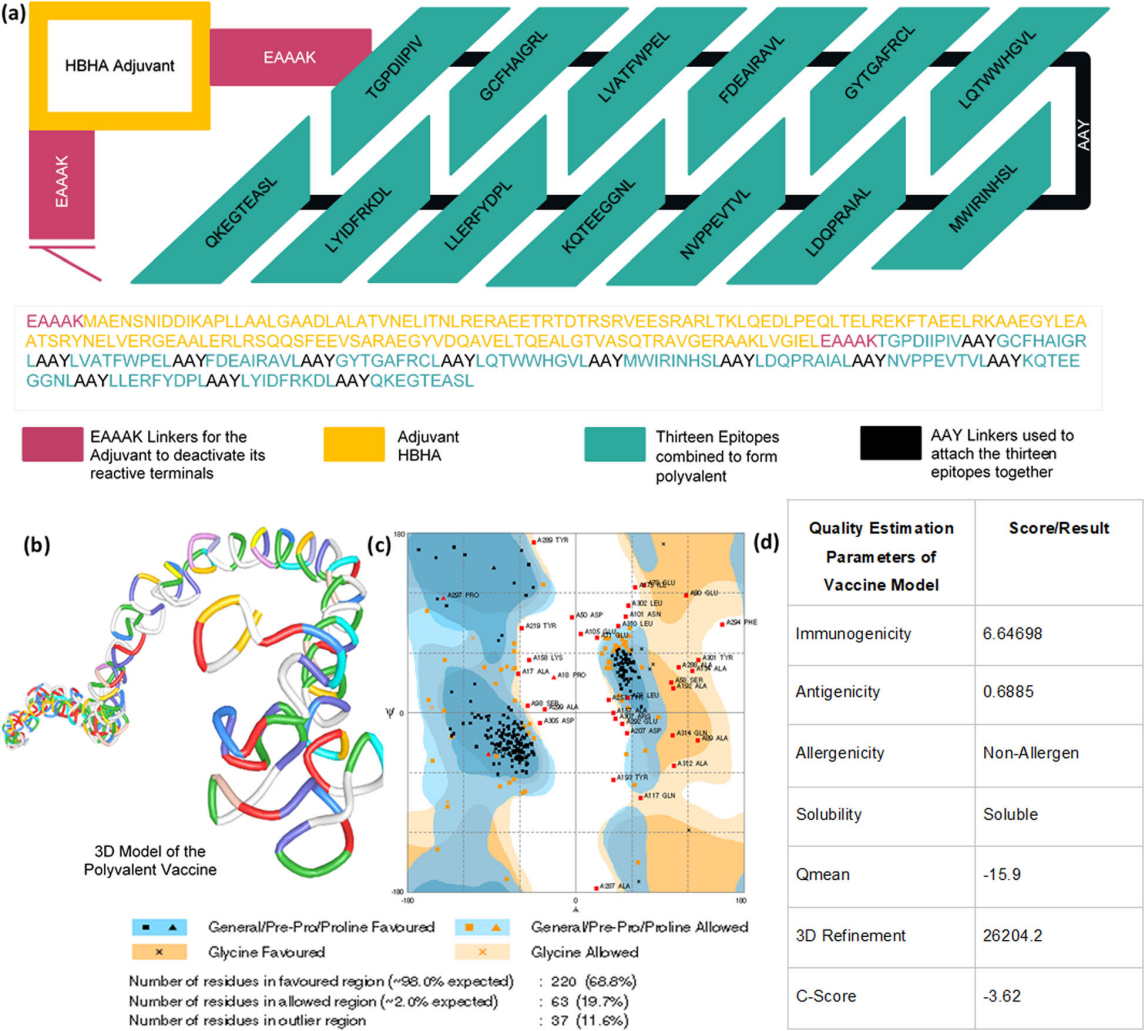
**a** Complete sequence of polyvalent vaccine showing the 13 epitopes joined by proper linkers AAY and the suitable adjuvant HBHA at the start, bordered by EAAAK linkers **b** generated polyvalent 3-D model **c** Ramchandaran Plot for quality estimation and configuration **d** Quality refinement and other quality parameters of polyvalent protein model

For the construction of final vaccine construct, predicted T-cell epitopes were linked to each other with help of suitable and flexible AAY linkers. HBHA (1–159 residues) as adjuvant was added at N-terminals of final constructs with the help of EAAAK linker (Fig. 9a). After adding linkers and adjuvant, the final construct contained 322 amino acid residues with the molecular weight of 35.6 kDa and 3D-model was generated (Fig. 9b). Our polyvalent epitope model showed significant antigenicity (> 0.5) determined by Vaxijen server. Analysis of Ramachandran plots indicated that 68.8% residues of polyvalent protein model (alpha-helices) were in the most favored regions with the 98% expectation while 2% in the allowed region. The Ramachandran plot is demonstrating low energy conformations for ϕ (phi) and (psi) angles of the model. The graphical representation shows the local backbone conformation of each residue and more than 68% residues are in favorable region (Fig. 9c). The model was refined and the quality of the construct was estimated by QMEAN score (− 15.9) and C-score (− 3.62) (Fig. 9d).

### Molecular docking: MHC class-I and epitope binding analysis

**Fig. 10:**
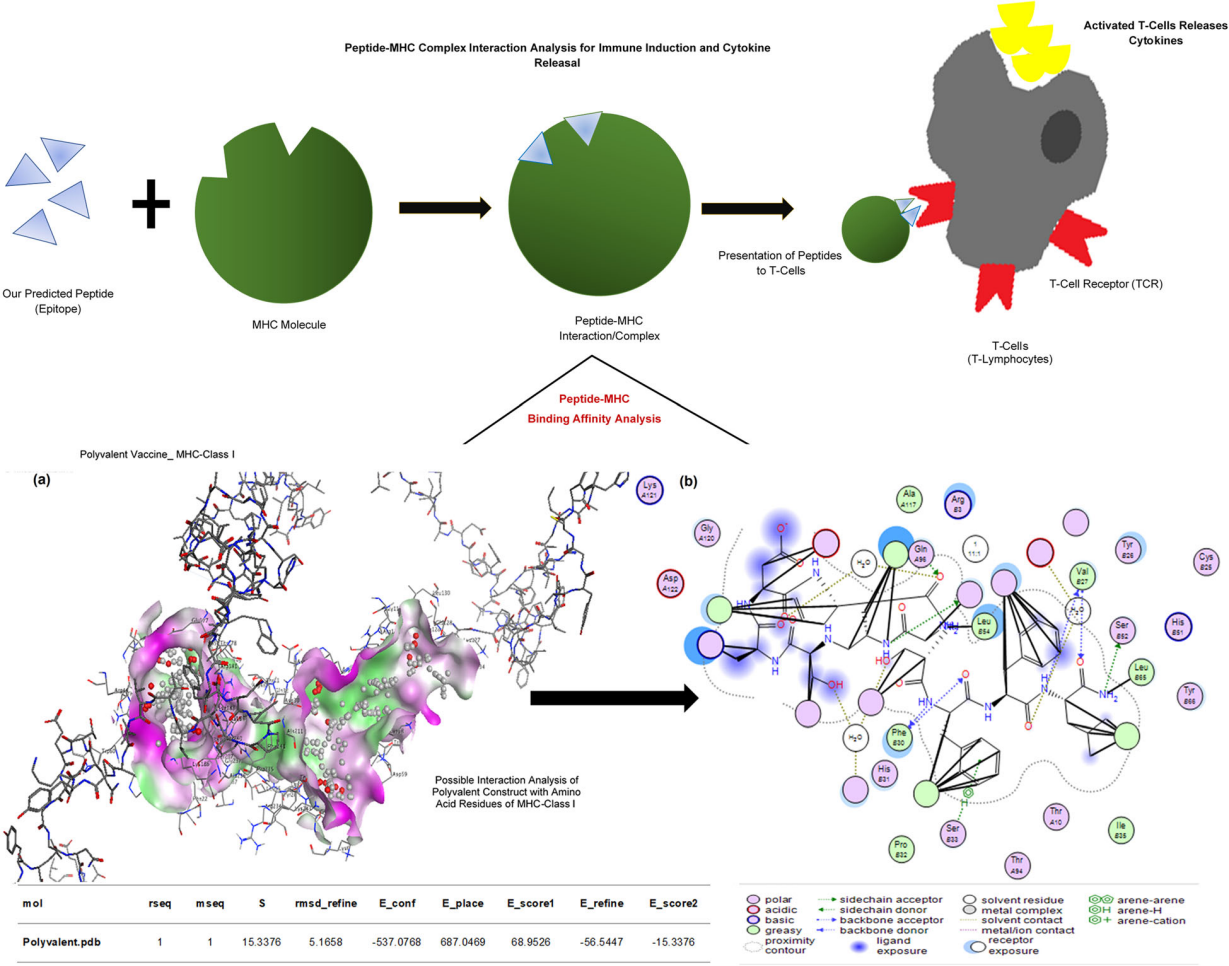
Activation of T-cells by peptide-MHC complex and cytokines production**.** In silico binding affinity analysis of T2DM polyvalent vaccine model with MHC-class molecule indicates optimum binding pattern (− 15.33 kcal/mol) **a** Molecular docking visual of polyvalent model with MHC class-I molecule **b** Potential interaction of polyvalent vaccine with amino acid residues of MHC class-I

We analyzed the binding affinity of polyvalent vaccine construct with MHC class-I molecule using MOE software. The lowest binding energies confirmed the optimum binding patterns between these molecules. The interaction of poly-epitopes (ligand) and MHC class-I (target) have been shown in Fig. 10a. We found the following binding energy of polyvalent construct with MHC class-I molecule: − 15.34 Kcal/mole (Illustration Table of Fig. 10a). It has been observed that polyvalent interacted with ARG, PRO, GLU, TRP, ASP, GLY, THR, LYS, ALA, PHE, TYR, GLN, MET, HIS and LEU amino acid residues of MHC class-I molecule (Fig. 10b). This interaction would prove that our polyvalent would be effective therapeutic vaccine candidate for T2DM.

## Discussion

Type 2 diabetes mellitus is a metabolic complex disease caused by several factors that is characterized by relative lack of obesity, insulin, insulin resistance, and high blood sugar [52, 53]. Although new therapies and management protocols to cure T2DM are being introduced, still the exact causes and effective treatment is imprecise. Currently, we are using different drug molecules to maintain blood glucose level and cure T2DM [54, 55]. These medications only minimize the symptoms of hyperglycemia and provide short term control. As a result, the drug resistance is developed in most of the diabetic patients and in the while, disease progress so fast that drugs alone cannot encounter it [56].

The current study signifies the importance of immunotherapies and vaccine development to modify the therapeutic strategies. Therapeutic vaccines could be effective to manage T2DM [57] as immune components have been found interactive with glucose transporter proteins [58].

It has been observed that the blood serum cytokines level elevates in diabetic conditions and in turn cytokines opens up the insulin independent glucose transporter channels and cells absorb the glucose [59]. The significant association of interleukin-1β (IL-1β) with T2DM is reported [53] and the role of antibodies has been studied in diabetic conditions [60].

The evidences about the association of GLUT-1 with immune components are available. It has been revealed that macrophages critically require GLUT-1 for their inflammatory response [61]. Also, the lymphocytes functioning demands high levels of energy that is assisted by GLUT-1 and in turn the expression of GLUT-1 remarkably increases with the increase of immune cells activation [17]. CD4 T-cells and T-effector cells also depends on GLUT-1 for their survival and programming regarding glucose metabolism and the GLUT-1 deficiency causes impairment of both glycolysis and T cells survival [15]. IL3 has reported to be involved in the trafficking and recycling of GLUT-1 intracellularly and controlling its expression on the cell surface, thus, maintaining the glucose uptake [62]. Cytokines like IL-1β enhances the expression of GLUT-1 and increase the glucose uptake in human articular chondrocytes [63].

Cytokines have also been known to affect and upregulate the GLUT-1 expression significantly by increasing the nitric oxide production levels [64]. Cytokines inhibit the tyrosine kinase activity which downregulates the insulin dependent GLUT-4 and upregulates the expression of insulin independent GLUT-1 [65]. CD28 alone as well as by secreting other immune mediators increases the GLUT-1 induction considerably [66]. TNFA (Tumor Necrosis Factor Alpha) has a cause and effect bond with GLUT-1 and plays a key role in the stabilization of GLUT-1 [67]. Similarly, stimulation of CD46 in CD4 T cells of humans causes the elevated expression of GLUT-1 on cells surfaces [68]. Numerous methods and databases for developing vaccines or immunotherapy against various pathogenic diseases have been developed over the last 20 years. T-cell epitope prediction methods that include indirect techniques such as Major Histocompatibility Complex prediction and transporter-associated protein binders [69]. Endogenous proteins may promote the hypercreativity and autoimmune reactions, however few studies showed that predicted epitopes of endogenous proteins exhibited significant, safe and effective results in animals. Recently, the vaccines for metabolic diseases have made considerable progress, particularly in the treatment of dyslipidemia, atherosclerosis, diabetes mellitus and hypertension, but comprehensive studies are required before any clinical applications [70]. Since T2DM is a multifactorial disorder, T2DM therapeutic-vaccine has been designed to predict obesity protein antigens [71]. Such vaccine targets include adipose tissue antigens, somatostatin, glucose-dependent insulinotropic polypeptide (GIP), and ghrelin [71]. In the studies of Zhang et al., 2018 and Zha et al., 2016 reported that cytokine L-1ß is a key proinflammatory substance in T2DM pathogenesis and has shown a reduction in weight gain, improved glucose tolerance and insulin sensitivity, and a lower β-cell loss in their vaccine with predicted peptide epitopes [55, 72]. Similarly, in phase I/II clinical trials vaccine Hil1bQb targeting IL-1β was found safe and well-tolerant [73]. Dipeptidyl peptidase 4 (DPP4) has been studied as an inhibitor of the glucagon-like peptide-1 (GLP-1) glucose-dependent insulinotropic peptide (GLP) [74], a therapeutic vaccine against DPP4 that has shown important, safe and successful results in the control of glucose levels in the mouse using GLP-1 [75]. *In-silico* method for the prediction of antigenic peptides and polyvalent construction could be used to develop vaccines for other human diseases. Molecular docking analysis of the refined vaccine structure with different MHC molecules and human immune TLR-2 receptor proved significant interaction [76]. In this case, the sequences of those proteins would be required against which we have to design a vaccine. This strategy could be effectively applied for the development of polyvalent vaccine candidates for other diseases specifically cancer and autoimmune diseases.

## Conclusion

This study focused to design potential T-cell poly-epitopes to simulate the immune components to provoke glucose transporters to control hyperglycemia. Our integrated immunoinformatic framework would strengthen the therapeutic discoveries and improve the treatment options to manage type 2 diabetes mellitus.

## Supplementary information

**Additional file 1.**

**Additional file 2.**

## Data Availability

The datasets generated during the current study have been included in manuscript and available as additional files.
